# Supplementary figures and images for: WIN 55,212-2 shows anti-inflammatory and survival properties in human iPSC-derived cardiomyocytes infected with SARS-CoV-2
Source: PeerJ. 2021 Oct 8;9:e12262. doi: 10.7717/peerj.12262 (PMC8504461; doi:10.7717/peerj.12262)

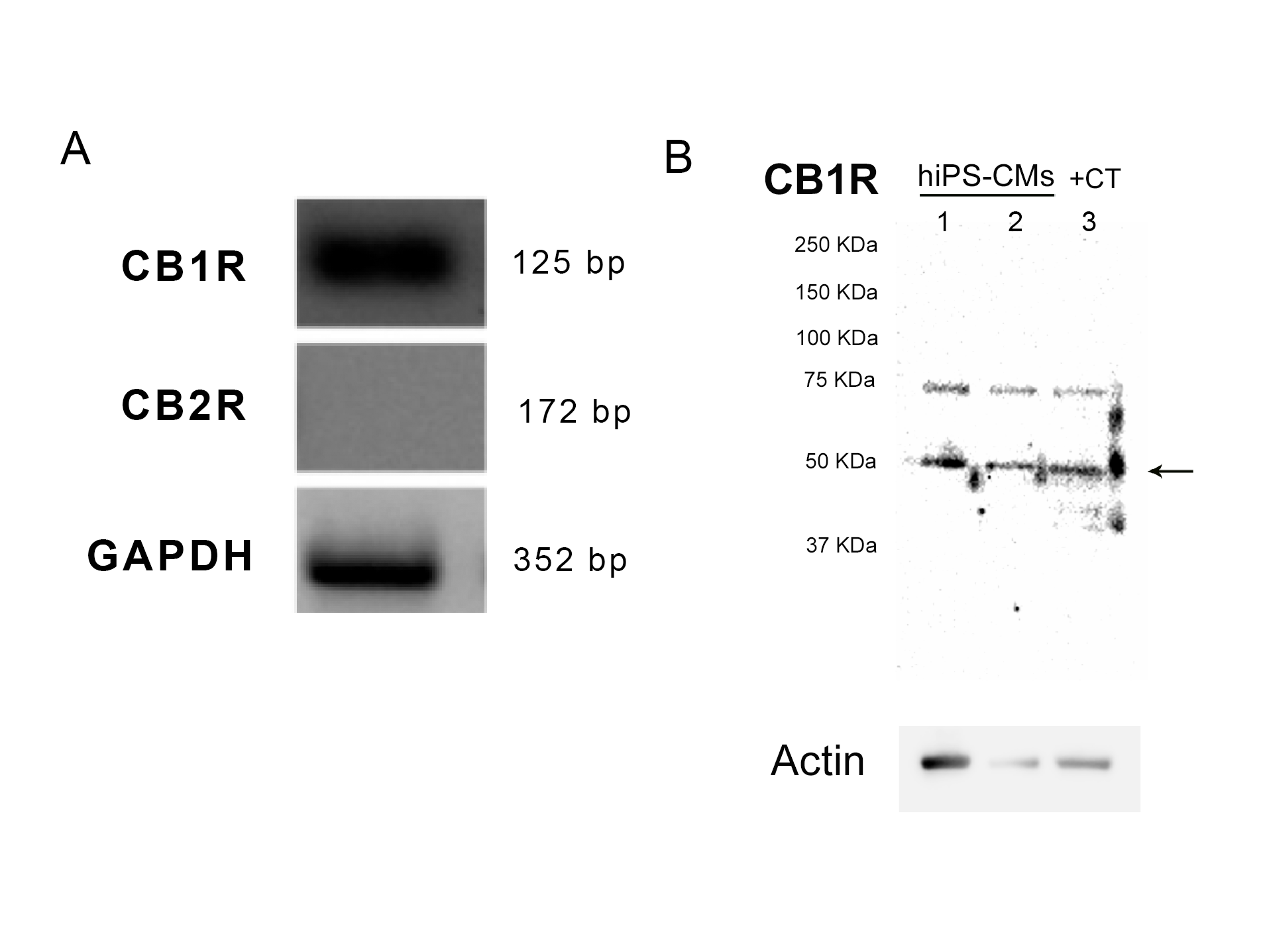

Supplement: Supplemental Information 1 — (A) The figure shows agarose gel of end-point PCR products for cDNA from hiPSC-CMs. One single specific band was detected for CB1 receptor (125 bp), while no such band was detected for CB2 receptor (172 bp). GAPDH (352 bp) was used as an endogenous control to confirm efficiency of the amplification reaction and quality of cDNA template. Data from two independent experiments. (B) Western blot detection of CB1 receptor protein levels in hiPSC-CMs. The arrows indicate the specific bands, corresponding to the molecular weight predicted to CB1 receptor. hiPSC-CMs samples were taken from different passages and the positive control (CT) was from tissue homogenates of adult Black C57/BL6 mouse hippocampus. [file peerj-09-12262-s001.png]

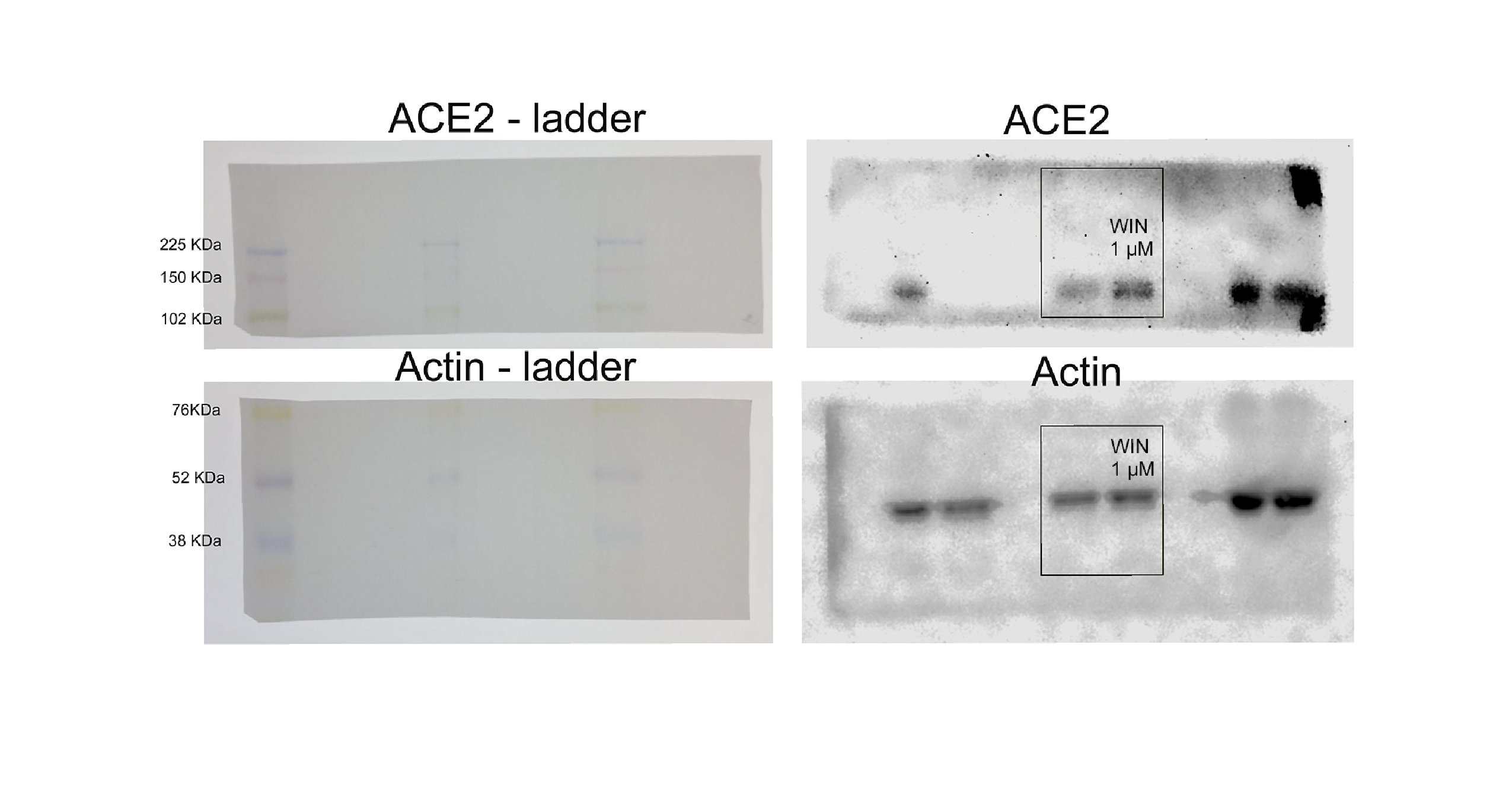

Supplement: Supplemental Information 2 — After transference the membrane was cut, the upper half used for ACE2 detection, and the bottom half used for actin detection. Images of the membranes used for detection with the ladder (Amersham ECL Rainbow Marker-Full range) are shown on the left. Full-length gels for ACE2 and actin with contrast adjusted to allow visualization of the membrane are shown on the right. Although the whole gel is shown here the lanes used for representative image in Fig. 1 are highlighted in a box. Other lanes have samples that are not related to the experiment in this manuscript. [file peerj-09-12262-s002.png]

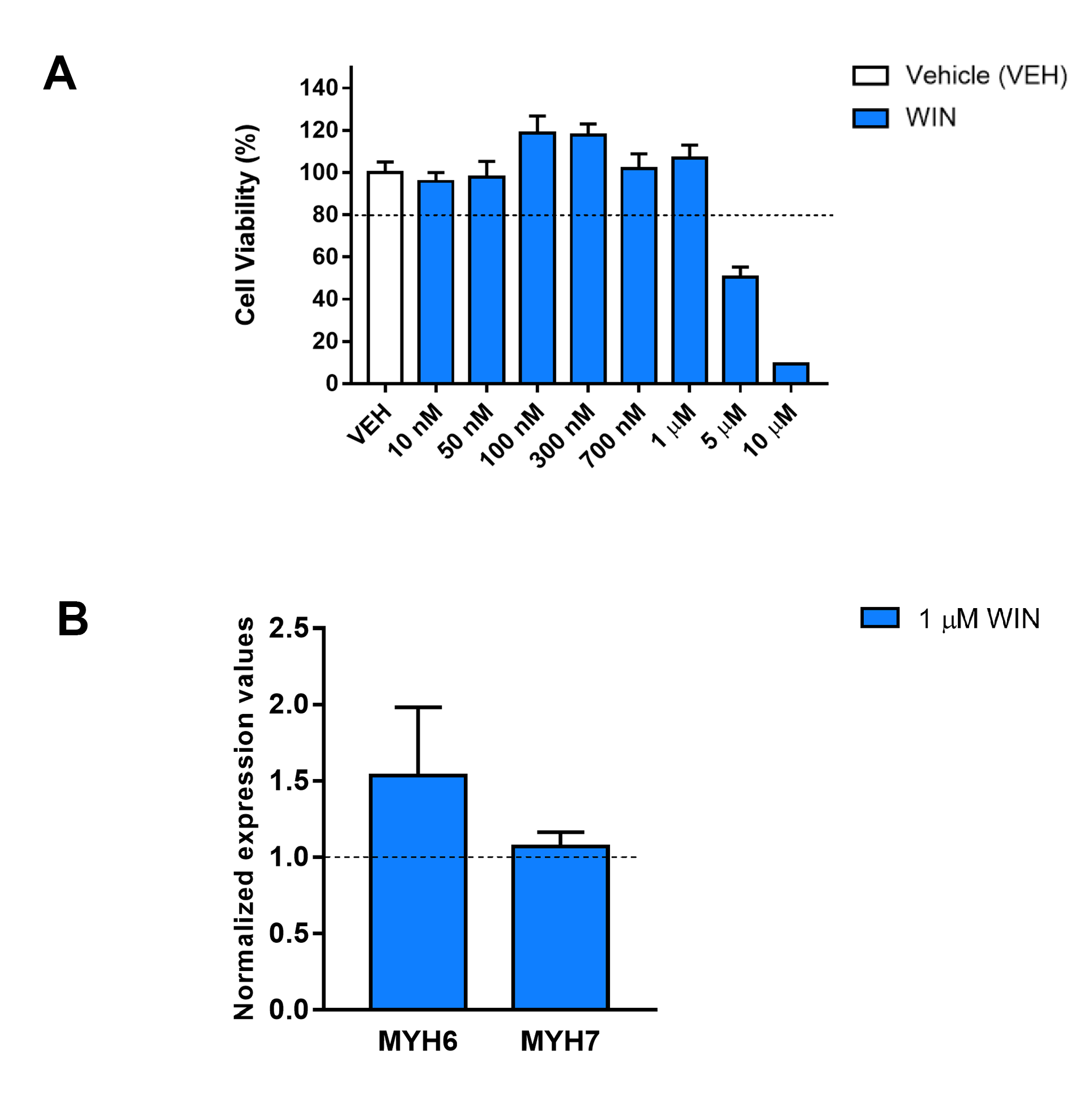

Supplement: Supplemental Information 3 — (A) Neutral red uptake assay from hiPSC-CMs treated with increasing concentrations of WIN for 72 h. The highest non-cytotoxic concentration was one μM. (B). qPCR for MYH6 and MYH7 genes from hiPSC-CMs treated with one μM WIN for 24 h. The MYH6 and MYH7 levels showed no significant differences between WIN-treated and untreated cells. [file peerj-09-12262-s003.png]

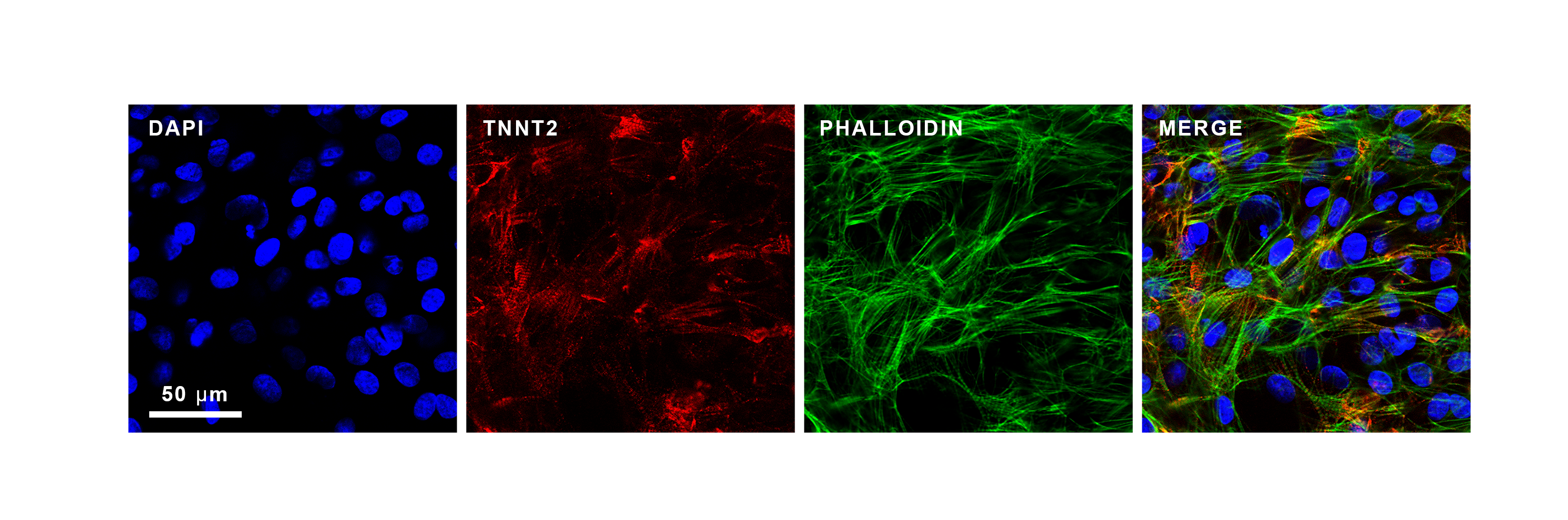

Supplement: Supplemental Information 4 — hiPSC-CMs were immunostained for TNNT2 (red), filamentous actin (F-actin) (green) by phalloidin staining and counterstained with DAPI (blue); 63 × magnification; Scale bar: 50 μm. [file peerj-09-12262-s004.png]
